# Polish Dairy Farm Transformations and Competitiveness 20 Years after Poland’s Accession to the European Union

**DOI:** 10.3390/ani14132013

**Published:** 2024-07-08

**Authors:** Wojciech Ziętara, Michał Pietrzak, Agata Malak-Rawlikowska

**Affiliations:** 1 The Institute of Agricultural and Food Economics, National Research Institute, 00-002 Warsaw, Poland; wojciech.zietara@ierigz.waw.pl; 2 Department of Economics and Organization of Enterprises, Institute of Economic Sciences, Warsaw University of Life Sciences—SGGW, 02-787 Warsaw, Poland; michal_pietrzak@sggw.edu.pl

**Keywords:** dairy sector, transformation, competitiveness index, labour productivity, farmland productivity, competitiveness of farms, Poland

## Abstract

**Simple Summary:**

This research aimed to assess the changes in Polish dairy production after EU accession (2004–2022) from the perspective of the sector’s competitiveness at the industry and farm levels. Polish milk production has experienced dynamic development, including a strong decline in the number of dairy farms and the number of cows, an increase in herd size, an increase in milk production, and growth in milk yield per cow. These were among the highest growth dynamics compared to other analysed countries. The scale of milk production is the basic factor determining the efficiency and competitiveness of dairy farms. Milk production is highly uncompetitive for smaller farms (<15 cows), as measured by the Corrected Competitiveness Index. Larger farms (with 30 cows and more) are competitive and responsible for the majority (~60–70%) of milk produced and delivered to the market.

**Abstract:**

Poland is one of the leading milk producers in the EU, being the fifth largest after countries such as Germany, France, Italy, and the Netherlands. From Poland’s accession to the European Union in 2004 up to 2022, Polish milk production experienced dynamic development. In this, there occurred a strong decline in the number of dairy farms (by −78%) and the number of cows (by −21%), an increase in dairy herd size (3.5 times) and increase in milk production (+60%) and milk yield per cow (by +62%). These were among the highest growth dynamics among the analysed countries. As a result of this significant transformation, Poland maintained an important position in milk exports, with a 31% export share in production in 2022. The scale of milk production was the basic factor determining the efficiency and competitiveness of dairy farms in Poland. Milk yield, farmland productivity, labour productivity, milk price, and the Corrected Competitiveness Index (based on labour and land opportunity costs) all showed a positive relationship with cow herd size on the farm. Milk production is highly uncompetitive for smaller farms (<15 cows). Despite substantial public support, the smaller farms, where subsidies equal up to 47% of total production value, could not earn sufficient income to cover the cost of capital, risk, and management in 2008, and even more so in 2021. This is because the farm income is too small to cover the extremely high opportunity cost of labour. The larger farms (with 30 cows and more) are competitive and responsible for the majority (~60–70%) of milk produced and delivered to the market. The most challenging from the sectoral policy point of view are medium farms (10–29 cows), whose share in production and deliveries is still important. To survive as economically viable units, these farms have to increase in scale and improve productivity. Otherwise, they will be gradually supplanted by larger farms.

## 1. Introduction

Poland is one of the leading milk producers in the EU, and it is the fifth largest after countries such as Germany, France, Italy, and the Netherlands. Since Poland’s accession to the European Union in 2004, Polish milk production has experienced dynamic development, significantly influencing its competitiveness against its European counterparts. Integration with the EU market not only opened up new export opportunities for Polish producers, but also caused a number of challenges related to adapting to European quality standards and changing agricultural policy [1]. The European Union support measures introduced within the Common Agricultural Policy have played a crucial role in restructuring Poland’s dairy sector by providing financial support for modernisation and efficiency improvements at the farm and processing level. These subsidies have enabled Polish dairy farms to invest in advanced technology and infrastructure, thereby enhancing production quality and competitiveness in the European market [2,3,4,5,6]. 

The issues of changes and developments in milk production and its competitiveness are discussed by some authors. While the changes in milk production in Poland are usually assessed as positive (e.g., a significant increase in the size of dairy farms, largedynamics of production growth, a substantial increase in the milk yield of cows), the problem of the efficiency and competitiveness of Polish farms is assessed ambiguously. This is because Poland is characterised by a rather fragmented, polarised structure of dairy farms of small size compared to the milk production leaders in the EU. The average Polish dairy farm had approximately 13 dairy cows in 2020, while herds in the largest milk-producing countries ranged from 54 in Italy to 101 cows in the Netherlands on average. Many authors point out that only larger farms achieve economic efficiency, and smaller farms have little chance of surviving in the liberalised EU market. Assessments of farm competitiveness depend on the adopted measures and the scope of assessment, but authors generally indicate the moderate competitiveness of Polish farms in relation to the leading producers and that the competitive position of Polish dairy farms has varied over time [7,8]. The competitive position is strongly diversified according to size of farms. Average-size and small dairy farms in Poland have the lowest production potential and weak competitive position, while larger dairy farms have a high competitive position, even in comparison with leading European producers of milk [8]. The main problem indicated by the authors is low labour productivity, especially on smaller farms [9,10,11]. Polish farms have significant resources in terms of their own labour, with a small share of hired workers. Due to the small scope of variability of labour resources, the input of this factor is constant and is not subject to optimisation [12]. The authors also point to the low technical efficiency, [13,14], the low cost efficiency of Polish dairy farms [12,15,16], and the weaker production potential of milk farms than in western and northern Europe [17]. They also indicate that production growth, especially in the first ten years after accession, was mainly due to input accumulation rather than productivity growth [13,15,18]. Despite all the challenges in Polish production, for 20 years of EU membership, the Polish dairy sector has retained its leading position in production as a significant exporter of dairy products, despite the changing market conditions and dynamic changes in policy support for this sector in the EU (including the liquidation of the milk quota system in 2015 and export subsidies in the EU).

In light of the abovementioned ambiguous results of the assessment of the competitiveness of dairy farms in Poland, this research aimed to assess the changes taking place in Polish dairy production after EU accession from the perspective of the competitiveness of the sector at the industry and farm levels. We characterise transformations and international competitiveness of dairy farming via the comparison of production parameters (production characteristics, export and self-sufficiency ratio) between the dairy sector in Poland and major producers in the European Union. To assess dairy farms’ competitiveness, we followed the concept of competitiveness proposed by Gallardo et al. [19], and later adopted by Kleinhanss [20]. They used a ratio of farm income divided by the opportunity cost of one’s own production factors at a farm (inputs), called the competitiveness index. In our assessments, we use a corrected version of this measure, based on labour and land opportunity costs. In addition to assessing competitiveness at the farm level, we investigated the root causes of the differences in competitiveness between different sizes of farm size groups. We performed an analysis of the productivity of production factors—land and labour in particular groups—that is not often found in the literature and can be treated as our additional contribution to the state of affairs. An additional contribution of this study is a comprehensive analysis that covers sector and farm characteristics over a long period of changes, starting from the 1990s through Poland’s accession to the EU in 2004 until 2022. Such cross-sectional analyses are rare due to the availability of data and the long comparison period. With this paper, we would like to fill this gap.

## 2. Conceptual Framework and Methodology

In this paper, we analyse the transformation of the main parameters characterising Polish dairy farms in the period 1996–2022, with particular emphasis on changes after Poland’s accession to the EU (2004). We use basic parameters for analysis, such as milk production, cow population and milk yield, the size and number of farms, and their competitiveness.

The competitiveness issue has attracted widespread interest among economists and management researchers. Nevertheless, there is no consensus on how to define this phenomenon and what methods to measure it with. One popular approach states that competitiveness is the ability to generate profits under competitive conditions. It can be assessed at the levels of nations, regions, industries, and companies (cf. [21]). Thus, the broadest approach to competitiveness is based on assessing it at the national level. According to the European Commission, the way to increase welfare is to be competitive at this level [22].

In our framework, we treat this as a starting point and call it Level 1 (see Table 1) or the macroeconomic level. Accordingly, one could also consider the meso-economic level, which can be seen from two perspectives. From the horizontal point of view, the nation’s competitiveness is built by the competitiveness of various geographical regions. From the vertical perspective, the nation’s competitiveness is determined by the competitiveness of different industries or sectors (cf. [21,23]). In our framework, we chose to use this second point of view—Level 2 in Table 1. We used Level 2 (industry competitiveness) to compare Poland’s dairy sector and top European Union producers. The selection of countries for comparison was purposeful. Germany, France, the Netherlands and Italy are among the leading milk producers in the EU. Austria has a farm structure similar to that of Poland, while the Czech Republic and Slovakia are Poland’s closest neighbours and were also part of the communist bloc and acceded to the EU simultaneously. In this comparison, we used very simple measures of competitiveness, such as a country’s share in EU production, farm size, milk yields, and sell-sufficiency ratios.

The competitiveness of a given industry or sector is built by the individual firms or farms, namely at the microeconomic level, which we call Level 3 in Table 1 (cf. [21,23]). The previous levels refer to aggregates. Level 3 refers to the actual decision-making units. At the micro level, competitiveness could be defined as a firm’s /farm’s ability to withstand competition while expanding its market share and generating profits. It involves offering more attractive deals than competitors. This encompasses creating, producing, and selling goods with more attractive prices and/or superior quality [23,24,25,26,27].

As depicted in our framework, competitiveness at the level of actual decision-making units stems from two main sources: enhancing firm productivity and efficiency and other factors (Which could include quality, which caters to customer needs, and external factors, e.g., market structure and regulations). According to Fischer and Schornberg [28], Latruffe [23], and Wijnands et al. [29], these first ones are crucial factors for shaping competitiveness at Level 3. Therefore, we focused on them in this paper. There are some authors that, similarly to us, conduct analyses of productivity/efficiency in agriculture and the agri-food sector (including dairy), for example, [6,23,30,31,32,33].

Productivity and efficiency are terms that are often used interchangeably, but they have slightly different meanings. Productivity refers to the relationship between the output volume and the volume of inputs in a given production process and can be expressed as a ratio, namely output(s) divided by input(s) [34]. As Latruffe [23] insightfully explains: “a general definition of productivity is the ability of production factors to produce the output. It can be simply measured as a partial productivity indicator, relating output to one input (e.g., yields or partial productivity of labour), but this does not account for the possibility of either factor substitution or output substitution.” (2010, p. 18). On the other hand, efficiency reflects the ability of a firm (or farm) to obtain maximum output from a given set of inputs [34]. Therefore, to be more efficient, any firm or farm has to experience higher productivity growth than its peers.

We measured the dairy farms’ competitiveness based on the ideas of Depperu and Cerrato [35] and Kleinhanss [20], which state that profitability is the most essential measure of competitive success. However, it is crucial to catch the category, which is the profit in the economic meaning, not just the accounting category. Accountants define profit as total revenues minus total costs. Economic profits “are the earnings after all costs—both money and implicit or opportunity costs—are subtracted” ([36], p. 295). In a world plagued by resource scarcity, any economic decision has an opportunity cost because choosing one way of conduct means giving up another way of conduct. “The opportunity cost is the value of the most valuable good or service forgone” ([36], p. 139). For example, any business firm usually owns a substantial part of its capital, and there is no accounting charge for the opportunity cost. Analogously, typically, a farm owns much of its farmland, while there is no accounting charge for the opportunity cost of this. Last but not least, in family farming, usually, a large part of the labour force is supplied by family members, while there is no accounting charge for the opportunity cost of this labour factor.

Therefore, we follow the concept of competitiveness measurement proposed by Gallardo et al. [19]. They used the ratio of farm income divided by the opportunity cost of their own production factors at a farm (inputs). Gallardo [19] called such a ratio the General Competitiveness Index, while Kleinhanss [20] uses a shortened name—the Competitiveness Index. The formula of this index is as follows ([19] p. 5; [20], p. 25–26):

$$CI_{f}=\frac{FNI_{f}}{OC_{w}+OC_{l}+OC_{c}}\begin{array}{c} >\\ =\\ < \end{array}1$$

where CI*_f_*—competitiveness index at a farm (f); FNI_f_—farm (f) net income; OC_w_—opportunity costs of work of family members; OC_l_—opportunity costs of one’s own agricultural land; and OC_c_—opportunity costs of one’s own capital.

However, based on the Polish Farm Accountancy Data Network (FADN) dataset, we were not able to calculate the methodologically correct competitive index for comparisons between years before 2014 and after that point. This was due to the substantial changes introduced in 2014 in the way the opportunity cost of one’s own capital is calculated. Furthermore, in 2016, some additional methodology modifications regarding the cost of one’s own capital were introduced. Therefore, it is impossible to perform reliable longitudinal comparisons in such a matter.

This is why we introduced the so-called corrected competitive index. The correction means that we do not take into account the opportunity costs of our own capital in the denominator of the index:

$$C{CI}_{f}=\frac{FNI_{f}}{OC_{w}+OC_{l}}\begin{array}{c} >\\ =\\ < \end{array}1$$

Of course, the above correction changes the interpretation of the index; however, it allows us to obtain reliable data and still deliver an informatively viable index of competitiveness. It informs us if a given farm can generate enough income to cover the opportunity costs of one’s own work and land. Let us return to the core idea of opportunity costs—choosing one thing means giving up something else. Farmers can find relatively easily alternative uses of their own land (by renting it) or their own labour (by hiring themselves somewhere outside the farm). The method proposed by us, CCI, does not consider the opportunity cost of one’s own capital; however, as Parzonko and Bórawski [7] noticed, sometimes important parts of a farm’s capital have no alternative use. Moreover, renting farmland or hiring oneself outside the farm could be quite easily reversible decisions, while alternative uses of the farm’s capital, even if available, often means selling the whole farm, which is an irreversible decision. Therefore, in our opinion, CCI is a valuable, informative index. It should not be treated as a substitution for the CI, but rather as complimentary. In the dataset available to us it is the only reliable option for longitudinal comparison.

We differentiate four classes of competitiveness based on the proposed CCI (which is in line with Kleinhanss’ proposition as regards CI ([20], p. 26)):

- CCI1—in the case of negative farm net income;
- CCI2—if partial coverage of opportunity costs of land and labour occurs, namely in the case when 0 ≤ CCI < 1;
- CCI3—if full coverage of opportunity costs of land and labour occurs, but when farm net income does not double up the such defined opportunity cost, namely in the case when 1 ≤ CCI < 2;
- CCI4—if full coverage of opportunity costs of land and labour occurs, and when farm net income at least double up the such defined opportunity cost, namely in the case when CCI ≥ 2.

**Figure 1:**
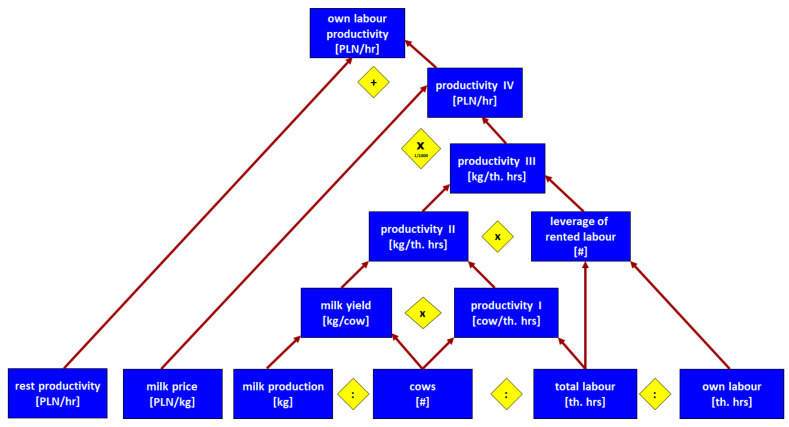
Pyramidal system of indicators building up own labour productivity of dairy farms. Note: arrows denote logic of arithmetical connections between indicators or other measures; diamonds denote the type of arithmetic operations between indicators or other measures. Source: own elaboration.

**Figure 2:**
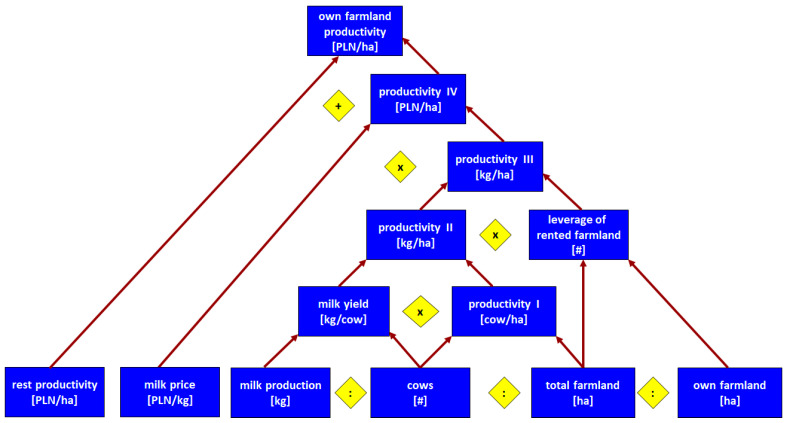
Pyramidal system of indicators building up farmland productivity of dairy farms. Note: arrows denote logic of arithmetical connections between indicators or other measures; diamonds denote the type of arithmetic operations between indicators or other measures. Source: own elaboration.

How is the value of the corrected competitive index built up? According to our framework from Table 1, competitiveness at the farm level depends on productivity, efficiency, and other factors (Level 4). In the present paper, we focused on the partial productivity of production factors. Regarding CCI construction, we focused on the productivity of own labour and the productivity of our own land. To explain in detail how particular groups of farms differ regarding productivity, we constructed pyramidal systems of indicators, building up labour productivity (Figure 1) and farmland productivity (Figure 2). In constructing those systems, we were inspired by the DuPont System of Financial Control [37], which we adapted to the farms and to the scope of our analysis.

Data used in our study were obtained from the following public databases:

- EUROSTAT (Statistical Office of European Union);
- The Central Statistical Office (in Poland);
- The Institute of Agricultural and Food Economics—National Research Institute database (Poland);
- Farm Accountancy Data Network Database—FADN (Poland).

## 3. Results

### 3.1. Changes in Milk Production, Number of Farms and Milk Yields

We characterise transformations in and the international competitiveness of the dairy farming industry (Level 2—compare Table 1) by comparing the production parameters (production, export, and self-sufficiency ratio) of the dairy sector in Poland and those of major producers in the European Union.

#### 3.1.1. Production and Export

**Figure 3:**
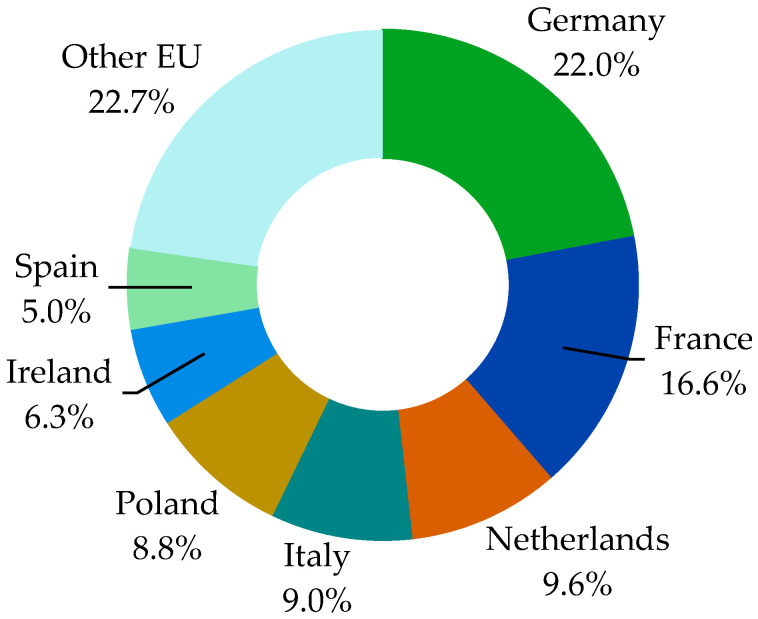
Structure of cows’ milk deliveries to dairies in the EU in 2022. Source: own elaboration based on Eurostat database [38].

Poland, with over 12.8 mln tones of milk collected in 2022 by dairies, is the fifth largest milk producer in the EU after Germany (31.9 mln tonnes), France (24.1 mln tones), the Netherlands (13.9 mln tonnes), and Italy (13 mln tones) [38]. Poland’s share in EU milk collection in 2022 was 8.8%. After joining the EU, milk production in Poland increased significantly (by 60%), as shown Figure 3, but Poland’s place among the leading milk-producing countries in the EU has not changed. Together, these 5 countries produce approximately 66% of the milk in the entire EU (expressed in milk deliveries to processing).

**Figure 4:**
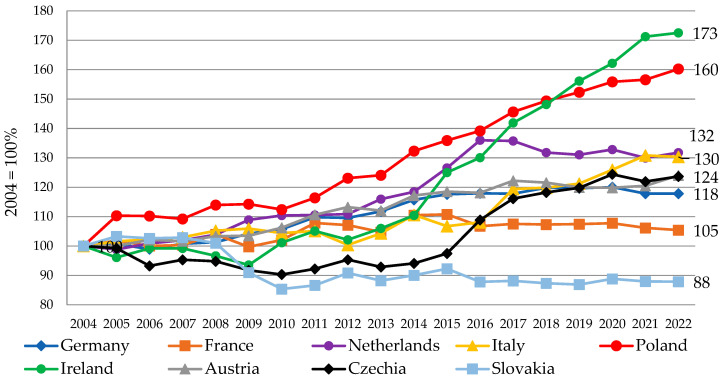
Dynamics of cow milk deliveries to dairies in Poland and main EU milk producers (2004 = 100%). Source: own elaboration based on Eurostat database [38], accessed 14 May 2024.

Despite the significant liberalisation of the milk market in the EU after 2015, including, first of all, the abolishment of the milk quota system (which limited EU milk production for over 30 years after 1984), as well as the abandonment of other forms of support such as subsidies for the export and private storage of dairy products, Poland was able to increase milk production significantly. The dynamics of cow milk deliveries to dairies in Poland and the main EU milk producers are presented in Figure 4. Compared to 2004 as a base, milk production in Poland has increased by 60%, including 24% growth after 2015. These were the strongest growth dynamics among the analysed countries, except for Ireland.

Milk production is an important component of agricultural market output in Poland. Table 2 presents numbers characterising the changes in the level of agricultural market output in Poland in the years 2005–2022. During this period, agricultural production increased by 3.3 times. Plant production increased slightly more, by 3.5 times, and animal production rose by 3.2 times. Despite the lower growth rate of animal production compared to plant production, it retained its dominant position in terms of agricultural market output. Its share in 2005 was 61.3%, and in 2022 it decreased to 59.4%. Milk production played an important role in animal production. Its share in 2005 was 32.2%, and in 2022 this was 34.2%. Milk production increased 3.4 times in the analysed period. Its growth rate was higher than the growth rate of commercial animal production.

Over the entire analysed period, Poland was self-sufficient in milk production. The milk production self-sufficiency ratio in Poland was calculated as the ratio of production to total consumption (including feed). In 2005, this amounted to 120%, and in all following years after 2015, it ranged from 119 to 123.9% [41,42,43,44]. This allowed Poland to maintain a strong export orientation. The relevant numbers related to the dairy trade are presented in Table 3. In the analysed years of 2005–2022, the export of milk and milk products from Poland in raw milk equivalent increased almost twice (by 96.4%), rising from 2435.4 thousand tons to 4785 thousand tonnes. During this period, the import of dairy products also increased significantly. Consequently, the positive balance of Polish foreign trade in raw material equivalent increased by 17.6%.

During the analysed period, the share of milk exports in milk production increased from 18.1% in 2005 to 31.3% in 2022, indicating significant potential for milk production in Poland. The results of foreign trade in milk and milk products are much more favourable regarding value (euro). In the analysed years, the value of milk and milk products exported increased fourfold, while the balance more than doubled (2.4). These results indicate that the exports are mainly dairy products with higher added value. This is the result of the efficiency of milk processing (dairy).

#### 3.1.2. Number of Cows, Farms and Milk Yields

**Figure 5:**
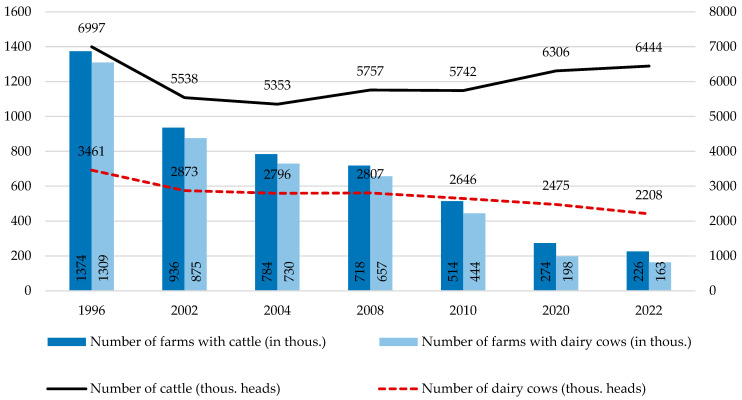
Number of dairy farms and the number of dairy cows in Poland in 1996–2022. Source: own elaboration based on [39,40,41,42,43,44].

During the period of the functioning of the communist system in Poland (until 1989), the so-called “planned economy” was characterised by the weakening of the operation of the market mechanism by establishing economic planning as a tool for organising the economy. The main goal of the economy was to maximise production. Concerning animal production, efforts were made to maintain high cattle, pig, and poultry populations. The largest cattle population in Poland in the post-war period amounted to 13,254 thousand units, including 6146 thousand cows, in 1975 [45]. Figure 5 shows the evolution of the cattle population, including dairy cows, and the number of farms breeding cattle, including those keeping dairy cows, from 1996 to 2022. In 1996, the cattle population amounted to approximately 7 million heads and was 47.2% smaller than in 1975, and the cow population was 3461 thousand heads. The decrease in the number of cows compared to the maximum in 1975 was 43.5%. The share of cows in the cattle population was 49.5%. From that year on, a systematic decline in the number of dairy cows was observed. At the time of Poland’s accession to the EU in 2004, the population of dairy cows was 2796 thousand heads. It decreased by 21% to 2208 thousand heads in 2022. In 2022, the share of dairy cows in the cattle population was 34.2% and was 18 percentage points (p.p.) lower than in 2004, and 15.3 p.p. lower than in 1996. The cattle population in this period, after a temporary decline of 20.8% in 2002, increased to 6444.1 thousand heads and was 7.9% lower than in 1996 and 20.4% higher than in 2004. This increase resulted from an increase in the population of beef cattle breeds [45].

Much greater changes occurred in the number of farms keeping cattle, including dairy cows (Figure 5). The number of farms with cattle breeding decreased from 1374 thousand in 1996 to 226 thousand in 2022. The decline was 83.6%, with the most significant decline occurring after EU accession (2004)—by 71.1%. The number of farms with dairy cows decreased to a greater extent, falling by 87.6% in the entire period 1996–2022, including by 77.8% since 2004. In 2022, there were 162.5 thousand dairy farms. To explain the main processes behind these changes, it is important to mention that farmers in Poland effectively resisted compulsory land collectivisation undertaken after World War II by the communist regime (repressive measures were introduced against individual farmers, such as the destruction of property, arrests, fines, and revisions). This resistance resulted in a unique ownership structure, where 76% of agricultural land in the late 1980s (just before the transition) belonged to individually owned farms [46,47]. In the dairy sector, the cooperative movement was one of the most important factors facilitating the changes during the economic system’s transition and during first years after Polish accession to the European Union [48]. Cooperative movements in Poland began in the 19th century. Before the transition, nearly 6.5 thousand agricultural cooperatives existed, but transformations led to a decline in their importance, especially in agriculture, where they were viewed negatively. However, dairy cooperatives remained successful, holding about 70% of the raw milk market share in 2004 [49]. After integration with the EU, Polish agriculture was covered by a number of instruments of the Common Agricultural Policy (e.g., the structural pension program and instruments supporting farm restructuring), which created opportunities for many farmers to leave the profession and hand over their farms to younger generations. The size of cattle farms was gradually increasing, and smaller farms, which did not follow economic pressures related to profitability and investments, changed their production profile to plant production or leased out land to larger entities [48,49,50,51,52]. Dairy processors (especially cooperatives) played a very important role in these processes, offering several incentive programmes to enable their suppliers to upgrade their production systems (for example, special premiums for high-quality milk, for farmers delivering larger quantities, or for having a cooling tank), extension services, and low-interest loans to enable farmers to invest [48].

During the analysed period of 2005–2020, the average size of the herd of dairy cows per farm increased by over 3.3 times, rising from 3.9 in 2005 to 12.7 in 2020 (Table 4). Despite such a significant increase, which was the largest among the countries presented in Table 4, there is still a considerable distance in relation to the average cow herds in selected European Union countries. The relevant numbers are given in Table 4.

The data in Table 4 indicate a significant distance between Polish dairy farms and farms in selected countries. In 2005, the cow herds in the EU-leading milk production countries were larger than those in Poland: in Germany, 9.8 times; in France, 9.6 times; in the Netherlands, 15.6 times; and in Italy, 7.8 times. In 2020, this distance slightly decreased, but it still remained substantial. At that time, cow herds in these countries were larger than Polish herds by 5.7, 4.9, 8.0, and 4.2 times, respectively. The distance between Polish dairy farms and Austrian ones was much smaller. In 2005, Austrian farms were about 2.5 times larger, and in 2020, only about 1.5 times larger. There is also a large distance between dairy farms in the Czech Republic and Slovakia. This distance results from the very “shallow” restructuring of farms in these countries after the change of the political and economic system in 1989, where large-scale farms in the form of agricultural cooperatives and state farms dominated. In Czechoslovakia, during the period of real socialism (until 1989), agriculture was dominated by large-scale farms operating in the legal form of agricultural production cooperatives and state farms. The share of family farms was very small. After the change of the political system in 1989, the existing farms changed their legal form, mainly to limited liability companies, in which the existing employees became partners. The organizational forms of these farms remained unchanged. The share of newly established family farms was negligible. After the political changes, the vast majority of these farms changed their legal form to cooperatives and commercial companies, remaining in an only slightly changed organisational form [53].

Regardless of the differences in terms of cow herd size, there is also a difference in the milk yield of cows between Polish farms and farms in selected countries. In 2005, the milk yield of cows on Polish farms was 4178 kg/year and was lower than that in Germany by 38%, France by 34%, the Netherlands by 45%, and Italy by 29%. In 2020, these differences were smaller: −13%; −1%; −18%; and +7%. The differences in relation to Austria, the Czech Republic and Slovakia in 2005 were 28%, 35%, and 27%, respectively. In 2020, the differences decreased significantly; the milk yield of cows on Polish farms was almost the same as that on Austrian farms and was lower than that on Slovak farms by 6% and that in the Czech Republic by 23%.

Similarly to the cow herd size per farm, Poland was characterised by the strongest dynamics of milk productivity growth among the surveyed countries. Between 2005 and 2020, this increase was 162%. It was related to progressive improvements in cow feeding (e.g., better feed quality and structure, applying TMR), advancements in production techniques (including sanitary–veterinary standards of production, increasing milking frequency, and switching from conventional to automatic milking systems), and change in dairy cow breed, with a progressive move to domination by the Holstein Friesian breed [54,55,56,57].

#### 3.1.3. Costs of Production

Poland has favourable environmental conditions for cattle breeding and the production of milk. The moderate, animal-friendly climate and the prevalence of lowland areas make it possible to cultivate the fodder plants (grasslands) used in cattle rearing [54]. Despite a decrease in the area of grassland, Poland experienced an increase in milk production, demonstrating the country’s ability to maintain and enhance milk production despite environmental changes [54]. In 2020, their area in Poland was 3202.82 thousand ha, of which the agricultural land area (UAA) shares 21.42% [58]. This is a very significant resource for ruminant breeding in Poland [45].

**Figure 6:**
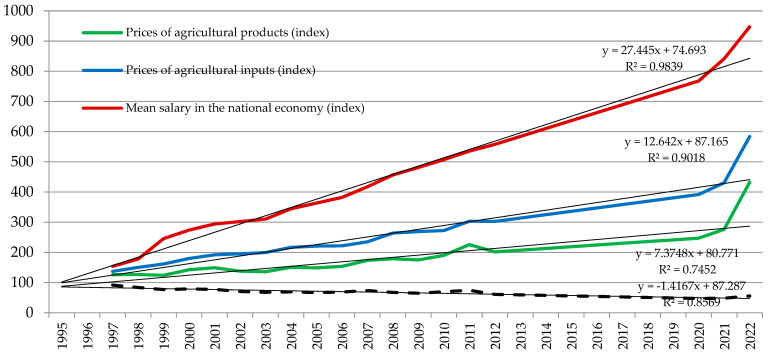
Trends in labor costs, agricultural production inputs and sales prices of agricultural products in Poland in the years of 1995–2022. Source: own elaboration based on [39,40,52].

Milk production depends on the economic conditions of agriculture, which constitutes an element of the national economy. There are specific trends in the level of labour costs in the national economy, the prices of production inputs for agriculture, and the sales prices of agricultural products. The main components of labour costs in the national economy are salaries. Their level affects labour costs in agriculture and the assessment of farmers’ income. Information about these trends is presented in Figure 6. In the analysed years 1995–2022, labour costs in the national economy increased more than 6 times, the prices of agricultural production inputs increased more than 4 times, and the sales prices of agricultural products only 3.4 times. The price increase was particularly pronounced in the years after the COVID-19 pandemic in 2020, being further worsened by Russia’s aggression against Ukraine in 2022 (Figure 6). Disruptions in supply chains, labour shortages, and increases in input prices (especially fertilisers and fuel) increased production costs dramatically. The pandemic also led to spikes in demand for certain agricultural products, exacerbating price volatility and contributing to overall inflation in the sector. These trends resulted in a decline in the unit profitability of agricultural production. A farmer who wanted to obtain income at a parity level (which corresponds to the average labour income obtained by employees in the national economy) was required to increase the production scale. This could be achieved by increasing the level of production intensity or by expanding the farm area and the number of animals kept. This economic pressure led to a reduction in the number of farms and in the concentration of production. The mentioned trends in the costs and prices of agricultural products occurred in all countries with a market economy.

### 3.2. Polish Dairy Farms’ Competitiveness by Size

#### 3.2.1. Corrected Competitiveness Index Results by Farm Size

We calculated the corrected competitive index (CCI) for the very first year when data from Polish FADN were available (namely 2008) and for the newest available data (namely 2021). Table 5 presents the results of this calculation, as well as all components of it. The results are split out between size categories used in Polish FADN as regards the dairy farms. We here used 7 categories to designate herd size in terms of milk cows:

(1)— below 5 cows, (3.6 cows on average);
(2)— from 5 to 9 cows, (7.5 cows on average);
(3)— from 10 to 14 cows, (12.6 cows on average);
(4)— from 15 to 19 cows, (17.4 cows on average);
(5)— from 20 to 29 cows, (24.5 cows on average);
(6)— from 30 to 39 cows; (34.7 cows on average);
(7)— 40 and more cows, (62.8 cows on average).

The average number of cows refers to 2021. It differed only slightly from the average cow numbers in 2008. There was the only one exception of the group (7), where the average number of cows was 54 in 2008.

Therefore, one could expect differences in income that were more or less proportional to the herd size and the opportunity cost of land and labour. But that was not the case. The opportunity costs of one’s own labour in the smallest farms were only around 1.6 times smaller than in the largest ones (in both years investigated), while the farm income was 19 times smaller in 2008 and nearly 26 times smaller in 2021. Thus, in group (1), a very small income has to cover the relatively high opportunity cost of one’s own labour, but it does not. Therefore, the CCI is much below one, which proves the lack of competitiveness in the smallest group. This is also the case of groups (2) and (3). When taking into account the opportunity cost of one’s own land, the situation in groups (1), (2), and (3) is even worse. Farms from group (4) could cover the opportunity cost of own labour by a very thin margin, one which was too low to cover the additional opportunity cost of land. Nevertheless, the situation of group (4) was better in 2021, when its CCI was above one, which was a sign of competitiveness (Table 5).

The groups (5)–(7) were competitive in both investigated years. Comparing group (4), which represents the medium size among Polish dairy farms, with the biggest group (7), one could find that (4) had only 1.2 times less cost of labour in both years 2008 and 2021, while more than 4 times less farm income (4.8 times in 2008 and 4.1 in 2021). Therefore, the situation was similar to that of group (1), but not as dramatic. Regarding land cost, the difference in 2008 was 2.4 times lower and improved in 2021 (2.1 times lower).

**Figure 7:**
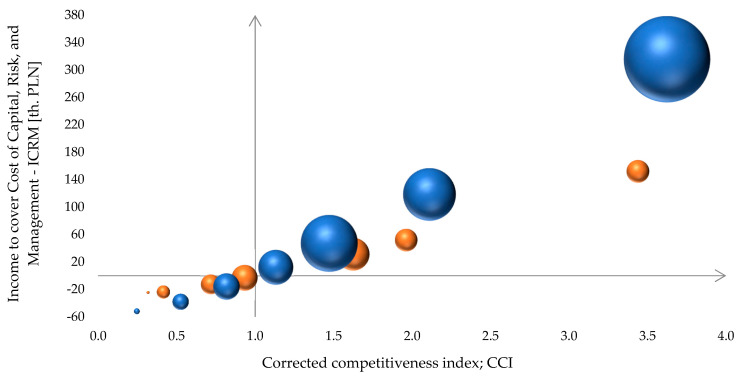
Corrected competitive index versus income to cover cost of capital, risk, and management by size groups of dairy farms in 2008 and 2021. Note: bubble size denotes the average milk production in respective groups; red colour denotes 2008, while blue colour denotes 2021. Source: own research based on [59,60].

Some additional details can be observed in Figure 7. The size of farms’ groups is represented by bubbles (red in 2008, and blue in 2021) from the left (group (1)) to the right (group (7)). The size of the bubbles represents the total milk production of all farms in a particular group; thus, it roughly reflects their role in the dairy sector in Poland. One could easily find that while, shortly after EU accession, the dominant role was played by groups (5) in the 20–29 cows range, the main role currently belongs to the biggest size category, (7) with 40+ cows. At the same time, group (1) was pretty marginal in both 2008 and 2021.

**Figure 8:**
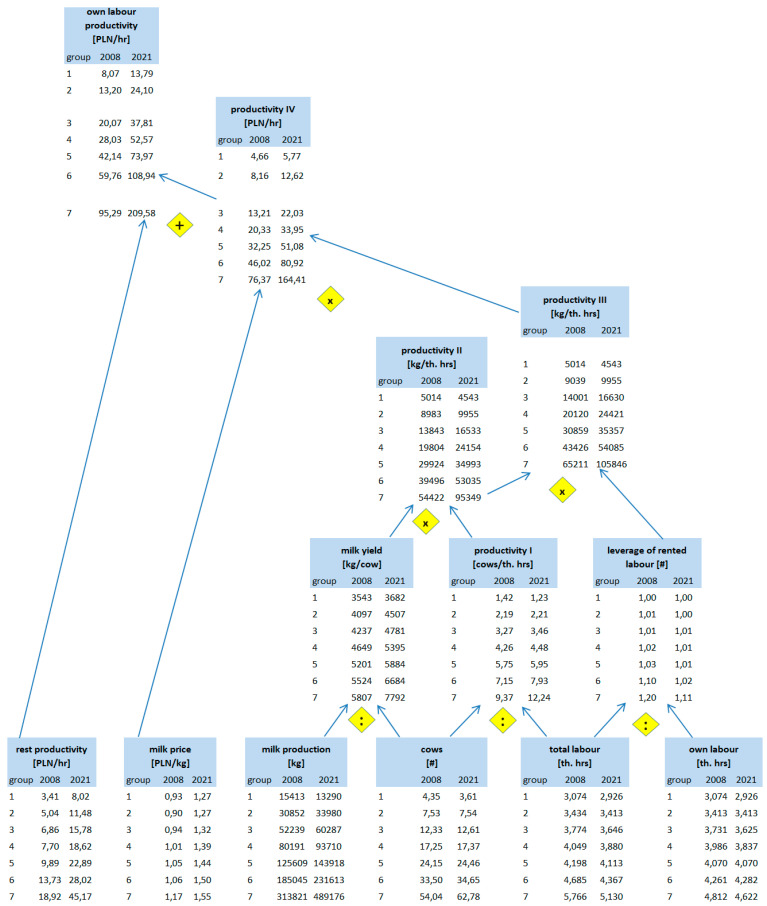
Pyramidal system of indicators explaining differences in labour productivity of dairy farms by size groups in 2008 and 2021. Source: own calculations based on [59,60].

However, these data refer only to the farms from the Polish FADN sample. The proportions in the whole dairy sector are not identical. According to [61], the number of farms keeping cows in Poland decreased from 730 to 155 thousand between 2005 and 2022, while the number of milk suppliers to milk processors fell from 320 to approximately 80 thousand, respectively. During the period in question, the share of the cow population of small farms with 1–9 cows (groups (1) and (2) in our classification) decreased from 49.7 to 11.5%. At the same time, the share of the cow population of medium farms with 10–29 cows (groups (3), (4) and (5) in our classification) increased slightly from 34.4 to 35.5%. Finally, the share of the cow population of larger farms with 30 or more cows (groups (6) and (7) in our classification) soared dramatically from 15.9 to 53.0% ([61] p. 9). Taking into account a clear correlation between herd size and milk yield per cow (cf. Figure 8), the disproportion of milk production between farm groups has to be even bigger than the share of the cow population. Thus, the differences in proportions between groups from the FADN sample—depicted in Figure 7—could not be very far from the reality of the whole population of dairy farms in Poland.

The groups are scattered across two variables: corrected competitive index and what we call income to cover the cost of capital, risk, and management (ICRM). ICRM is the Farm Income less opportunity costs of own labour and own farmland. It should then cover the opportunity cost of own capital, and the risk premium as well as the management effort of the farmer. Figure 7 visualises the distances between farm groups across these two variables, as well as how they changed from 2008 to 2021. It is worth noticing that the production of milk is highly uncompetitive at the smallest farms, where it is performed at relatively small scale; thus the impact of highly competitive larger farms on the general sector picture is much more important. Smaller dairy farms are still a challenge, particularly from the social policy point of view; however, they are not a crucial part of the Polish dairy sector landscape.

More equivocal are medium farms from the groups (3), (4), and (5). Again it is worth noticing the switching position of group (4), which was uncompetitive in 2008 but changed its classification in 2021. Group (3) was uncompetitive in both years under investigation; however, its CCI improved from 0.72 to 0.82. Group (5) was competitive both in 2008 and 2021; however, it is declining in terms of CCI. The larger farms from the groups (6) and (7) are highly competitive. The CCI is around 2 in the first case and around 3.5 in the second one. In both cases, the CCI improved slightly from 2008 to 2021.

The groups differ regarding public support (which comes mainly from EU Common Agricultural Policy). The largest group received 7.9 times larger subsidies than the smallest one in 2008. The analogous relation was 5.2 in 2021. However, the differences in total production value were much more substantial, namely 18.5 and 24, respectively. Therefore, the relation of subsidies to total production value was 33% in group (1), while it was only 14% in group (7) in 2008. In 2021, the differences were even bigger: 47% and 10%, respectively. One could easily find that dependency on subsidies substantially increased in the smaller farms, where subsidies were equivalent to one third (group (2)) and even to nearly half (group (1)) of total production value. In the larger farms, it varied between 10 and 15%, while in medium farms it varied between 19 and 29% (Table 6).

Despite substantial public support, the smaller farms could not earn what we call income to cover the cost of capital, risk, and management (ICRM) in 2008, and this was even more pronounced in 2021. This was because the farm income was too small to cover the extremely high opportunity cost of labour. In the case of a group (1), the issue was so severe that the loss calculated at the ICRM level was as big as the total production value in 2008, and in 2021 this loss was equivalent to 129% of the total production value. This occurred despite high subsidies, without which the loss would be equivalent to 175% of the total production value. In the case of a group (2), analogous figures were 46% and 80%, respectively. The larger farms would achieve positive ICRM values even without subsidies. However, even in the second largest groups with 30–39 cows, ICRM would drop substantially, falling by 59% without subsidies. In group (7), cutting subsidies would decrease ICRM by slightly less than one third (Table 6).

Again, more equivocal are medium farms from the groups (3), (4) and (5). Group (3) noticed a loss at the ICRM level in both years under investigation, despite subsidies. Group (4) developed from a small loss at the ICRM level in 2008 into a positive ICRM in 2021. However, it would not be able to earn positive ICRM if subsidies were cut. Group (5) in 2008 was able to earn positive ICRM, even without receiving subsidies; however, it lost this ability in 2021 (Table 6).

#### 3.2.2. Root Causes of the Differences in Corrected Competitiveness Index between Farms

To investigate the root causes of the differences in competitiveness between farm size groups, we performed an analysis of production factors, such as land and labour, and how productively they are used in particular groups. As we already know, the biggest difference between groups regards the opportunity cost of labour; thus, let us start with this production factor. Table 7 depicts the differences in one’s own and rented labour and the total labour force. The smallest farms employ only 1.9 (2008) and 1.8 (2021) times smaller labour forces than the largest ones. When taking into account only one’s own labour disparity squeezes (due to the nonexistence of a rented workforce on the smallest farm), it was only 1.6 in both years under investigation. Even in medium farms (groups (3)–(5)) and farms from group (6), the role of the rented workforce is quite marginal. In the biggest farms, rented labour accounted for 17% in 2008 and 10% of the total labour force in 2021. Except for group (1), the intensity of the engagement of family members of working age in work on the farm (measured by hours per person) increased between 2008 and 2021 (Table 7).

As regards total farmland area (cf. Table 8), group (1) was 8.9 (in 2008) and 5 (in 2021) times smaller than in group (7). Even the smallest farms’ group use rented land. The proportion of rent to the total farmland area was stable between 2008 and 2021. It accounted for 12% of the smallest farms, while on the biggest farms it was 3 times larger (around 36%). While in group (1), family members of working age working on the farm dealt with around 3.6 (in 2008) and 5.2 (in 2021) hectares on average, in group (7), the farm family members of working age working on the farm dealt with around 15.5 hectares in both years under investigation.

To assess the productivity of own labour force and own land, we used the pyramidal systems of indicators introduced in the conceptual framework (Chapter 2). Figure 8 depicts how the own labour productivity ratio [PLN/hr] is built up. As we already know, the difference in labour force, as measured by the difference in working hours between groups (7) and (1), was only 1.9 (2008) and 1.8 (2021). However, when we took into account how these working hours translate into the number of cows the labour force was able to deal with, the differences increased to 6.6 and 10, respectively. This was the first sign of substantial differences in labour productivity according to farm scale. At this level, we used the term of productivity I (Figure 8).

These differences are further reinforced by the advantages of larger farms in terms of milk yield. Milk yield increased in all categories of farms; however, the increments were bigger in larger farms. While cows from the largest farms yielded 1.6 more milk than those from smaller ones in 2008, thirteen years later, such differences increased to 2.1. As a result, the advantage of larger farms is even bigger on the productivity II level than on the productivity I level. In 2008, on the largest farms, one thousand hours of work was involved, resulting in a 10.9 times bigger milk volume than on the smallest one. In 2021, this advantage is as 95,349 to 4543 kg of milk per th. h; namely, group (7) outperforms group (1) 21 times (Figure 8).

When we consider “the leverage” of rented labour, the advantage will be multiplied further. In most farms, rented labour does not exist, or it is used only in small amounts. Thus, the leverage is 1 or very close to it. In the largest group, this leverage is noticeable, and therefore, it multiplies the results of group (7). At the level of productivity III (kilograms of milk volume per thousand hours of own work), the largest farms outperformed the smallest ones by 13 and 23.3 times in 2008 and 2021, respectively (Figure 8).

Another area of difference driven by farm scale is the price received for the milk produced. The price advantage of the largest farms against the smaller ones decreased from 1.3 to 1.2 between 2008 and 2021, but is still noticeable. Multiplying kilograms of milk volume obtained per one thousand hours of one’s own work by the milk price and factor 1000 to adjust the units, we obtain productivity IV, namely, the milk value per one hour of own work. It counts for 4.66 (2008) and 5.77 (2021) PLN per hour in the smallest farms, while in the largest ones, it counts for 76.37 and 164.41 PLN per hour, respectively. Therefore, group (7) outperforms group (1) by 16.4 and 31.3 times respectively. At this level of productivity, the advantage of the largest farms over the smallest ones is the biggest (Figure 8).

The farms investigated specialised in milk production; however, this does not mean they do not benefit the yield of other forms of farm production. We divided the total production value by the value of milk production by one’s own labour force, as measured by hours. We called such a ratio “rest productivity of own labour”. It should be added to productivity IV to receive the final ratio of “own labour total productivity”. It counts for 8.07 (2008) and 13.79 (2021) PLN per hour in the smallest farms, while in the largest ones, it counts for 95.29 and 209.58 PLN per hour, respectively. Therefore, group (7) outperforms group (1) by 11.8 and 15.2 times, respectively. One could find that, on this level, the advantage of the largest farms is still huge, but less than on level IV of productivity. This is according to the relatively better results of the smallest farms in other types of agricultural production than milk (Figure 8).

**Figure 9:**
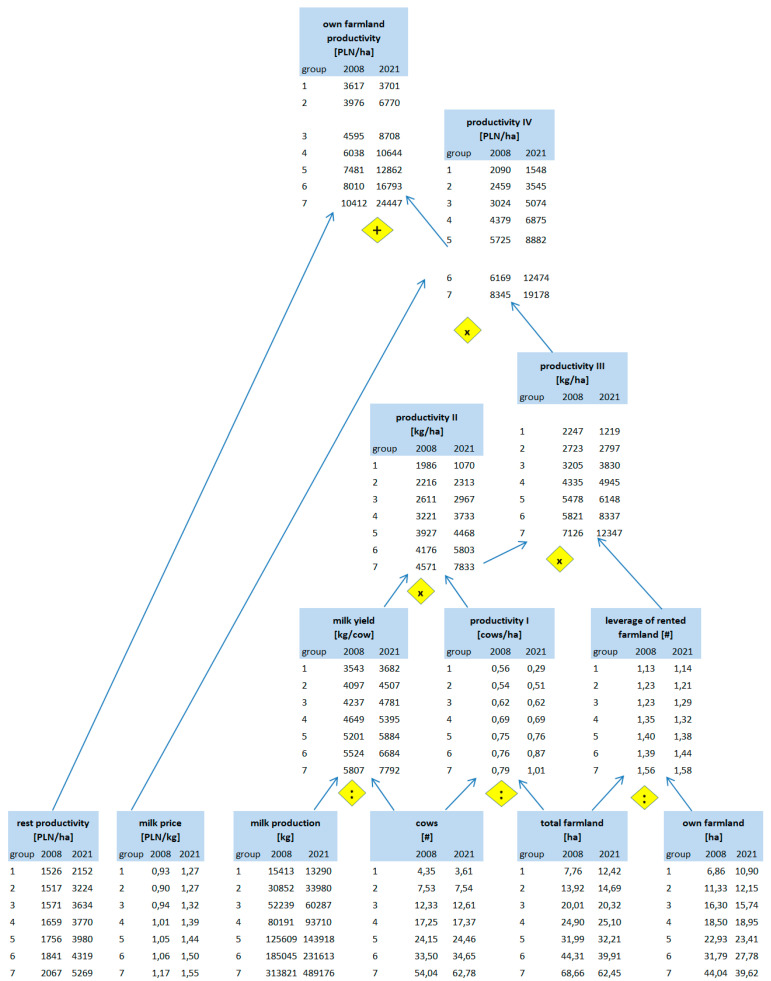
Pyramidal system of indicators explaining differences in farmland productivity of dairy farms by size groups in 2008 and 2021. Source: own calculations based on [59,60].

The next assessment area is the productivity of one’s own farmland and we again used the pyramidal systems of indicators introduced in the conceptual framework (Chapter 2). Figure 9 depicts how the own farmland productivity ratio [PLN/ha] is built up. As was already mentioned, the difference in total farmland between groups (7) and (1) was 8.9 in 2008 and 5 times (in 2021). However, the differences in cow numbers are bigger—14.4 and 17.4 times, respectively. Therefore, when we take into account the coverage, namely cows per hectare (productivity I), we found that the largest farms outperformed the smallest one, exceeding it by 1.4 times in 2008 and 3.8 times in 2021 (Figure 9).

As we already know, larger farms have an advantage in terms of milk yield. These differences multiply the edge of larger farms in productivity I. As a result, the advantage of larger farms is even bigger on the productivity II level. In 2008, the largest farms obtained 4571 kg of milk from every hectare, compared to 1986 kg in the smallest ones (2.3 times more). In 2021, this disproportion was even more striking, namely 7833 versus 1070 kg per hectare (7.3 times more—Figure 9.

When we take into account “the leverage” of rented farmland, the advantage will be multiplied further. Farms from every group rent farmland; thus, the leverage is higher than 1 for every group. Nevertheless, this leverage is biggest in the largest farms (around 1.4 times bigger than in the smallest ones in both years of investigation). The leverage ratio multiplies productivity II; thus, the gain in milk volume per hectare is larger when only taking into account one’s own land (productivity III), as compared to the total farmland. The largest farms outperformed the smallest ones by 3.2 and 10.1 times in 2008 and 2021, respectively (Figure 9).

As mentioned, group (7) has a price advantage over group (1). Multiplying kilograms of milk volume obtained per hectare of one’s own farmland by the milk price results in a productivity IV ratio, namely, the milk value per one hectare of own farmland. It counts for 2090 (2008) and 1548 (2021) PLN per hectare in group (1), while in group (7) it counts for 8345 and 19,178 PLN per hectare, respectively. Therefore, the largest farms outperform the smallest ones by 4 and 12.4 times, respectively. At this level of productivity, the advantage of the largest farms over the smallest ones is the biggest (Figure 9).

The farms under investigation benefit from other forms of farm production outside of milk production. We divided the total production value minus the value of milk production by one’s own farmland as measured by hectares. We called such a ratio the “rest productivity of own farmland”. It should be added to productivity IV to receive the final ratio of “own farmland total productivity”. It counts for 3617 (2008) and 3701 (2021) PLN per hectare in the smallest farms, while in the largest ones, it counts for 10,412 and 24,447 PLN per hectare, respectively. Thus, finally, the group of largest farms outperforms the group of the smallest counterparts by 2.9 and 6.6 times, respectively. Similarly to the productivity of one’s own labour, the smaller advantage of the group (7) at this level of productivity occurs due to the relatively better results of the smallest farms in other types of agricultural production than milk (Figure 9).

To sum up, the productivity of the two investigated production factors, namely labour and farmland, differ by farm size. Larger farms can use land and labour resources more efficiently. The aspect of production technology cannot be underestimated here, even though it was not the subject of our analyses. However, it should be mentioned that the profitability of investments and the possibility of implementing technological solutions increases with the size of the farm and its investment potential, which also depends on the size.

Our results indicate that larger farms outperform the smaller ones. While this advantage is already substantial in terms of farmland, it is dramatic in the case of labour. As it is illustrated in Table 9, the value of production per hectare is at least 7 (up to 41.3) bigger than the opportunity cost of one hectare of one’s own farmland. On the other hand, only in farms with 15 cows and more is the value of production at least twice as big as the opportunity cost of one’s own labour. In group (2), this ratio is only slightly above 1, while in group (1), this ratio is below 1, which means the value of the whole production is not able to cover labour costs alone.

Therefore, it is clear that milk production on small farms does not make sense from an economic point of view. Small farms (groups (1) and (2)) have around a 10% share of milk production in Poland, while their share in milk deliveries to the dairy industry is marginal. Larger farms (groups (6) and (7)) are competitive and they are responsible for the majority of milk produced and delivered to the market.

The most challenging of these from the sectoral policy point of view are the medium farms (groups (3), (4), (5)), whose share in production and deliveries is still important. To survive as economically viable units, these medium-sized farms have to increase in scale and improve productivity (Table 9). There are many solutions that can be used to improve the productivity of medium-sized farms. Most of them are related to production techniques and technology [62]. The introduction of precision livestock farming technologies can significantly enhance farm management efficiency, animal health, and overall productivity [60]. However, these types of investments require a larger scale of production to become profitable. For example, rumination collars provide data that can help to detect health issues early [63,64]. Another solution could be to optimise feeding strategies [65] using, for example, alternative protein sources [66] and alternatives to traditional starch sources [67] to help mitigate the risks associated with feed supply and price volatility, improve the nutritional profile of dairy cattle diets, and improve overall herd management. Another important aspect is effective health monitoring to ensure the animals’ well-being, including avoiding heat stress, which is nowadays a trigger factor in dairy farms and is related to observed climate change [68,69]. We could name more examples of farm productivity improvements [70], but this would not be efficient. It is because Polish farms have significant resources of their own labour, with a small share of hired workers. These own labour resources are not subject to optimisation at the farm level; thus, it is difficult to improve labour productivity, even with technical improvements.

## 4. Summary and Conclusions

Poland is one of the leading milk producers in the EU, and it is the fifth largest after countries such as Germany, France, Italy, and the Netherlands. Since Poland’s accession to the European Union in 2004, Polish milk production has experienced dynamic development. Integration with the EU market opened new export opportunities for Polish producers, but also posed challenges in adapting to European quality standards and agricultural policies. EU support measures within the Common Agricultural Policy provided crucial financial aid for modernisation and efficiency improvements, enabling investments in advanced technology and infrastructure to enhance production quality and competitiveness. Thus, this research aimed to assess the changes taking place in Polish dairy production after EU accession from the perspective of the sector’s competitiveness at the industry and farm levels.

The changing socio-political conditions, including the progressive liberalisation of the European Union’s agricultural policy and its opening to global dynamics, as well as the progressive spatial integration of markets between Poland and the European Union, resulted in a higher growth rate of labour costs in the national economy and the prices of agricultural inputs than the sales prices of agricultural products. This resulted in a decline in the unit profitability of agricultural production. These trends sped up the processes of concentration and specialisation in milk production. Their effect was a strong decline in the number of dairy farms (by −78%) and the number of cows kept (by −21%). As a result, the average size of the cow herd increased from 3.9 in 2005 to 13.6 in 2022. Despite this increase, the average size of the cow herd in Poland was several times smaller than the average cow herd in other leading milk-producing countries. Regardless of the decline in the number of dairy farms, milk collection by dairies in 2022 amounted to 12.8 million tons and was 60% higher than in 2004. This resulted in an increase in cows’ milk yield by 62%. This was one of the highest growth dynamics compared to the analysed countries. Since 2005, the self-sufficiency ratio in Poland is around 120%, and the share of milk exports in milk production increased from 18.1% in 2005 to 31.3% in 2022.

Addressing the main research purpose of this paper, which was assessing competitiveness at the farms’ level, and the root causes of the differences in competitiveness between different farms’ size groups, we found that the scale of milk production was the basic factor determining the efficiency and competitiveness of dairy farms. The following factors were positively related to the number of cows: cow milk yield, farmland productivity, labour productivity, milk purchase price, and competitiveness index. The production of milk is highly uncompetitive at smaller farms (having less than 15 cows) and is relatively small; thus, the impact of highly competitive larger farms (with more than 30 cows) on the general sectoral picture is much more important. Despite substantial public support, the smaller farms could not earn sufficient income to cover the cost of capital, risk, and management (ICRM) in 2008, and this even more severe in 2021. This is because the farm income is too small to cover the extremely high opportunity cost of labour. Dependency on subsidies substantially increased in the smaller farms, with subsidies equalling up to one third and even one half of total production value. The larger farms would achieve positive ICRM even without subsidies, both in 2008 and in 2021.

To investigate the root causes of the differences in competitiveness between farm size groups, we performed an analysis of production factors, such as land and labour, and how productively they are used in particular groups. The productivity of labour and farmland differ by farm size. Larger farms outperform smaller ones. While this advantage is already substantial in terms of farmland, it is dramatic in the case of labour. Only in farms with 15 cows and more is the production value at least twice as big as the opportunity cost of one’s own labour.

Therefore, it is clear that milk production does not make sense from an economic point of view on small farms. The existence of these farms results from the social problem of human resources being captive in agriculture production after the Polish transition from communism into a capitalistic system. Thus, this problem should be solved or mitigated through social policy, not a policy that addresses the problems of the sector. The smaller farms (<10 cows) have around a 10% share of milk production in Poland, while their share in milk deliveries to the dairy industry is marginal (~3–5%). The larger farms (with 30 cows and more) are competitive and are responsible for the majority (~60–70%) of milk produced and delivered to the market. The most challenging issue from the sectoral policy point of view are the medium-sized farms (10–29 cows), whose share in production and deliveries is still important. To survive as economically viable units, these kinds of farms have to increase in scale and improve productivity. Otherwise, they will be gradually supplanted by larger farms.

One should remember about some limitations of our study. They relate to the competitiveness measures of Gallardo [19] and Kleinhanss [20], which consider the farm’s production scale and quality of resources. To partly address this limitation, the cost of capital was omitted from our analysis of opportunity costs as this was assumed to be irreplaceable at the farm level. Future research could focus on the impact of policy changes on the competitiveness of Polish dairy farms and the role of technological progress in this process. A comparative analysis with more EU countries would also be valuable.

## Figures and Tables

**Table 1:** Conceptual framework—levels of considering competitiveness phenomenon.

Levels	Structure of Factors			
Level 1	Nation’s competitiveness			
Level 2	industry competitiveness			other factors
Level 3	firm or farm competitiveness		other factors	
Level 4	firm or farm productivity and efficiency	other factors		

Source: own adaptation based on [23].

**Table 2:** Agricultural market output in Poland during the period 2005–2022.

Specification	Years				
	2005	2010	2015	2020	2022
Agricultural market output total [mln PLN]	42,907.0	59,357.1	74,202.7	92,499.5	143,049.7
Index (2005 = 100)	100.0	138.3	172.9	215.6	333.4
Crop production [mln PLN]	16,605.6	26,116.3	30,815.4	37,670.0	58,037.2
Index (2005 = 100)	100.0	157.3	185.6	226.9	349.5
Animal production [mln PLN]	26,301.4	33,240.8	43,387.3	54,828.7	85,012.4
Index (2005 = 100)	100.0	126.4	165.0	208.5	323.2
Cows’ milk output [mln PLN]	8475.3	10,691.1	12,212.4	16,911.4	29,088.9
Index (2005 = 100)	100.0	126.1	144.1	199.5	343.2
Share of animal output in total output. [%]	61.3	56.0	58.5	58.8	59.4
Share of cows’ milk output in animal output [%]	32.2	32.1	28.1	30.8	34.2

Source: own elaboration based on [39,40].

**Table 3:** Foreign trade in dairy products in Poland in 2005–2022.

Specification	Years				
	2005	2010	2015	2020	2022
In the volume of milk equivalent [thousand tones]					
Export	2435.4	2314.2	3984.0	4730.0	4785.0
Import	259.0	870.6	1587.0	2100.0	2225.0
Net saldo	2176.4	1443.6	2397.0	2630.0	2560.0
Share of export in production (%)	18.1	16.9	30.2	31.8	31.3
Self-sufficiency ratio (%)	120.1%	112.6	122.2	121.5	120.2
In value [mln euro]					
Export	899.6	1208.1	1650.4	2316.9	3611.3
Import	146.9	430.3	793.9	1113.7	1774.3
Net saldo	752.7	777.8	856.5	1203.2	1837.0
Index of export in value, 2005 = 100	100.0	134.3	183.4	257.5	401.4
Index of trade saldo in value, 2005 = 100	100.0	103.3	113.8	159.8	244.1

Source: own elaboration based on [41,42,43,44].

**Table 4:** Average size of dairy herd per farm and milk yield in selected European Union countries in 2005–2020.

Countries		Size of Dairy Cow Herd per Farm (Units)			Index 2005 = 100	Av. Milk Yield per Cow kg/Year		Index 2005 = 100
	2005	2010	2016	2020		2005	2020	
Germany	38.4	46.4	61.8	72.4	188.7	6717	8853	116
France	37.4	45.0	57.1	62.0	165.9	6353	6860	108
Netherlands	60.9	74.6	97.5	101.3	166.3	7567	8323	110
Italy	30.5	35.2	37.7	53.7	176.1	5900	6324	107
Poland	3.9	5.9	9.0	12.7	325.6	4178	6788	162
Austria	9.8	11.3	17.6	19.3	197.1	5811	6784	117
Czech Rep.	65.0	122.9	128.6	158.1	243.3	6385	8853	139
Slovakia	14.4	24.5	30.9	39.3	274.1	5693	7188	126

Source: own calculations based on Eurostat data (database on number of dairy farms, dairy cow numbers and total raw milk production on farms), accessed on 14 May 2024.

**Table 5:** Polish dairy farms’ competitiveness index and categories used to calculate it by size.

Group by Cows Number	2008				2021			
	Farm Income [th. PLN]	Opportunity Cost of Own Labour [th. PLN]	Opportunity Cost of Own Land [th. PLN]	Corrected Competitiveness Index	Farm Income [th. PLN]	Opportunity Cost of Own Labour [th. PLN]	Opportunity Cost of Own Land [th. PLN]	Corrected Competitiveness Index
(1) <5	11.28	34.27	1.33	0.32	16.85	62.93	5.74	0.25
(2) 5–9	16.75	38.17	2.20	0.41	41.86	73.41	6.40	0.52
(3) 10–14	32.09	41.60	3.16	0.72	70.72	78.22	8.30	0.82
(4) 15–19	44.96	44.58	3.59	0.93	105.18	82.96	9.99	1.13
(5) 20–29	81.16	45.52	4.45	1.62	147.14	87.62	12.34	1.47
(6) 30–39	105.76	47.73	6.17	1.96	225.25	92.10	14.64	2.11
(7) ≥40	214.36	53.80	8.54	3.44	435.94	99.41	20.88	3.62

Source: own calculations based on [59,60].

**Table 6:** Total production value, subsidies and income to cover the cost of capital, risk, and management (ICRM) in Polish dairy farms by size.

Farm Groups by Number of Cows	2008				2021			
	Total Production [th. PLN]	Subsidies [th. PLN]	Subsidies [% of Production]	ICRM * [th. PLN]	Total Production [th. PLN]	Subsidies [th. PLN]	Subsidies [% of Production]	ICRM * [th. PLN]
(1) <5	24.8	8.1	33%	−24.3	40.3	18.8	47%	−51.8
(2) 5–9	45.1	13.3	29%	−23.6	82.3	28.2	34%	−38.0
(3) 10–14	74.9	17.6	24%	−12.7	137.1	39.4	29%	−15.8
(4) 15–19	111.7	21.7	19%	−3.2	201.7	45.5	23%	12.2
(5) 20–29	171.5	27.5	16%	31.2	301.1	58.6	19%	47.2
(6) 30–39	254.6	35.4	14%	51.9	466.5	70.3	15%	118.5
(7) ≥40	458.6	64.4	14%	152.0	968.6	98.1	10%	315.7

* ICRM is the Farm Income less opportunity costs of own labour and own farmland. Source: own calculations based on [59,60].

**Table 7:** Labour force in Polish dairy farms—by size.

Farm Groups by Number of Cows	2008				2021			
	Total Labour [h]	Rented Labour [h]	Own Labour [h]	Own Labour [h/Person *]	Total Labour [h]	Rented Labour [h]	Own Labour [h]	Own Labour [h/Person *]
(1) <5	3074	0	3074	1593	2926	0	2926	1407
(2) 5–9	3434	21	3413	1504	3413	0	3413	1544
(3) 10–14	3774	42	3731	1548	3646	21	3625	1626
(4) 15–19	4049	64	3986	1601	3880	42	3837	1705
(5) 20–29	4198	127	4070	1590	4113	42	4070	1754
(6) 30–39	4685	424	4261	1658	4367	85	4282	1784
(7) ≥40	5766	954	4812	1683	5130	509	4622	1812

* Members of family being of working age and working on farm. Source: own calculations based on [59,60].

**Table 8:** Farmland in Polish dairy farms—by size.

Farm Group by Number of Cows	2008				2021			
	Total Farmland [ha]	Rented Farmland [ha]	Own Farmland [ha]	Own Farmland per Person [ha/Person *]	Total Farmland [ha]	Rented Farmland [ha]	Own Farmland [ha]	Own Farmland per Person [ha/Person *]
(1) <5	7.76	0.90	6.86	3.55	12.42	1.52	10.90	5.24
(2) 5–9	13.92	2.59	11.33	4.99	14.69	2.54	12.15	5.50
(3) 10–14	20.01	3.71	16.30	6.76	20.32	4.58	15.74	7.06
(4) 15–19	24.90	6.40	18.50	7.43	25.10	6.15	18.95	8.42
(5) 20–29	31.99	9.06	22.93	8.96	32.21	8.80	23.41	10.09
(6) 30–39	44.31	12.52	31.79	12.37	39.91	12.13	27.78	11.58
(7) ≥40	68.66	24.62	44.04	15.40	62.45	22.83	39.62	15.54

* Members of family being of working age and working on farm. Source: own calculations based on [59,60].

**Table 9:** The role of farms’ groups and their ability to cover the opportunity cost of labour and farmland.

Group by Cows Number	Estimated Share (2021)		Own Labour Productivity vs. Opportunity Cost of Labour		Own Farmland Productivity vs. Opportunity Cost of Farmland	
	In Milk Production	In Milk Deliveries to Dairies	2008	2021	2008	2021
(1) <5	~10%	~3–5%	0.7	0.6	18.6	7.0
(2) 5–9			1.2	1.1	20.5	12.8
(3) 10–14	~30%	~25–27%	1.8	1.8	23.7	16.5
(4) 15–19			2.5	2.4	31.1	20.2
(5) 20–29			3.8	3.4	38.6	24.4
(6) 30–39	~60%	~70%	5.3	5.1	41.3	31.9
(7) ≥40			8.5	9.7	53.7	46.4

Note: the opportunity cost of own labour was 11.2 PLN/hr in 2008, and 21.5 PLN/hr in 2021; the opportunity cost of one’s own farmland was 194 PLN/ha in 2008, and 527 PLN/hr in 2021. Source: own calculations and estimations based on [59,60].

## Data Availability

Data were derived from the public resources available in the public domains mentioned in the References.
